# "This is the first time I felt they're mine": the significance of tattoos in coping and identity reconstruction following mastectomy

**DOI:** 10.1007/s00520-026-11144-5

**Published:** 2026-08-27

**Authors:** Tsipi Hanalis-Miller, Avital Genkin, Avital Gershfeld-Litvin

**Affiliations:** The Academic College of Tel Aviv-Yaffo, Tel Aviv-Yaffo, Israel

**Keywords:** Breast cancer, Mastectomy tattoos, Grief, Body image, Coping mechanism, Meaning-making, Identity reconstruction

## Abstract

**Background:**

Breast cancer and subsequent mastectomy result in significant physical and emotional changes, including damaged body image, compromised sense of femininity, diminished self-esteem, and profound experiences of grief and loss. While reconstruction procedures are routinely offered, some women choose artistic mastectomy tattoos as an alternative means of reclaiming their bodies and reconstructing identity.

**Objective:**

Grounded in the Dual Process Model of Coping with Bereavement, this study explored how women utilize post-mastectomy tattoos as coping mechanisms and meaning-making processes following breast removal.

**Methods:**

A qualitative study combining thematic analysis of semistructured interviews with social semiotic analysis of mastectomy tattoos. Twelve women (mean age 44.5 years, range 30–56) who underwent mastectomy: 11 followed by breast reconstruction and one with a flat closure, and subsequently chose mastectomy tattoos participated in interviews conducted June 2023–April 2024. Data were analyzed to identify emerging themes and visual symbolism.

**Results:**

Three primary themes emerged: (1) bodily perceptions post-surgery—characterized by damaged body image, feelings of alienation, compromised femininity and wholeness, and struggles with intimacy; (2) mastectomy tattoos as coping mechanisms—involving restoration-focused planning and meaning-making processes; and (3) mastectomy tattoos as reclaimed identity, fostering renewed sense of belonging, confidence, and bodily control. Semiotic analysis revealed tattoos symbolized personal growth and transformation, freedom, and reconnection to nature and life.

**Conclusions:**

Mastectomy tattoos serve dual functions within the grief process: facilitating confrontation with loss while enabling restoration-oriented coping and identity reconstruction. Findings support incorporating artistic tattooing into post-mastectomy care options and expanding insurance coverage.

**Supplementary Information:**

The online version contains supplementary material available at https://doi.org/10.1007/s00520-026-11144-5.

## Introduction

### Breast cancer, mastectomy, and body image

Breast cancer remains the most common cancer among women globally, affecting over 2 million women in 2022 alone [1]. For many women, treatment includes mastectomy—the surgical removal of the entire breast [2]. While these procedures can be lifesaving, they result in significant bodily alterations that, combined with social and cultural expectations surrounding the female form, lead to profound emotional and psychological challenges [3].

The physical consequences of breast removal extend beyond visible scarring. Women face breast asymmetry, nipple removal, decreased nerve sensitivity leaving the breast area numb to touch post-surgery, and, in a substantial proportion of cases, chronic post-mastectomy pain syndrome [4–6]. These changes compel patients to confront not only physical transformations but also challenges to their sense of identity, femininity, and sexuality. Research consistently demonstrates that following breast removal surgery, women experience decreased feelings of sexual attractiveness, body dissatisfaction, and emotional distress including depression and anxiety [3, 7, 8]. For many, the discomfort extends to intimate relationships, with women reporting difficulty being seen or touched after surgery, contributing to feelings of unattractiveness [8].

### Current post-mastectomy options and their limitations

Contemporary medical practice typically offers breast reconstruction procedures aimed at restoring what has been lost. These options include breast and nipple reconstruction, prosthetics, and medical nipple tattoos [1, 8]. However, these procedures cannot fully restore the breast’s functional and sensory properties. Smooth muscle tissue and lactiferous ducts—the pathways through which milk flows during breastfeeding—cannot be reconstructed, affecting some women’s connection to maternal identity [7, 9]. Moreover, reconstructed breasts frequently lack sensation, compounding the sense of bodily alienation many women experience post-surgery.

Post-mastectomy tattooing broadly encompasses two distinct practices. Nipple-areola complex (NAC) tattooing is a reconstructive, medically oriented procedure aimed at recreating the visual appearance of a natural nipple and areola, typically performed by a trained medical tattooist or clinician as a final step in breast reconstruction [10]. In contrast, decorative artistic tattooing, the focus of the present study, involves personalized artwork applied over or around the mastectomy site, prioritizing symbolic and aesthetic expression rather than anatomical replication. The two approaches are not mutually exclusive and are sometimes combined [10], but they differ in purpose, technique, and the meanings women attribute to them.

While surgeons routinely discuss these cosmetic options with patients, an emerging alternative: artistic mastectomy tattoos, is typically not suggested by medical professionals, despite being widely promoted through media channels and patient advocacy groups [10]. This pattern is not universal, however: In some clinical settings, physicians, nurses, or plastic surgeons perform NAC tattooing directly or refer patients to certified medical tattoo artists as part of standard reconstructive care [10]. Nonetheless, outcomes may be compromised when specialized artistic techniques—such as three-dimensional rendering and color matching—are not available, which can contribute to psychosocial distress. Unlike medical nipple tattoos that aim to replicate lost anatomy, artistic mastectomy tattoos transform surgical scars into personalized artwork, offering women an opportunity to reclaim their bodies through creative expression rather than attempted restoration of the precancer body.

### Mastectomy tattoos: an emerging phenomenon

The practice of marking the body to commemorate significant life events has deep historical roots. Traditional societies have long incorporated body modifications to express grief and mark transitions, such as the tongue tattoos practiced among Aboriginal Hawaiians following the death of significant individuals [11]. These practices recognize embodiment—the manifestation of experiences through physical form—as encompassing emotional, physical, mental, and social states [12].

In contemporary contexts, tattoos serve multiple functions beyond aesthetic decoration. Women have historically utilized tattoos to challenge restrictive social roles and reclaim bodily autonomy following traumatic experiences including illness and abuse [13]. More recently, women recovering from breast cancer have employed tattoos to redefine mastectomy scar aesthetics while articulating their cancer journey and subsequent recovery [13, 14]. Through body adornment, women may experience empowerment by reclaiming aspects of themselves that were lost or violated—a process identified as central to recovery from trauma more broadly [15]. Studies indicate that women who choose mastectomy tattoos after surgical recovery demonstrate positive increases in body image and self-esteem [16]. Tattoos appear to help women alter perceptions of their bodies, regain control, and foster positive changes in their interactions with the world [17]. For many women with breast cancer, tattoos help shape identity in relation to illness, symbolizing resilience and strength against physical and social consequences of cancer [18].

### Theoretical framework: coping and meaning-making after loss

Understanding mastectomy tattoos as coping mechanisms requires situating them within established frameworks of grief and loss. Stroebe and Schut’s [19, 20] Dual Process Model of Coping with Bereavement offers a valuable lens for examining how women navigate the profound losses associated with mastectomy. This model emphasizes two complementary processes: loss-oriented coping, which involves directly confronting and processing the emotional impact of loss, and restoration-oriented coping, which focuses on adapting to new reality and rebuilding meaning. Effective coping involves oscillating between these two processes—allowing oneself to grieve while simultaneously creating new goals and finding ways forward.

Mastectomy represents a complex form of loss that extends beyond physical tissue removal. Women grieve not only the loss of breasts as body parts but also the symbolic meanings attached to them: femininity, sexuality, maternal capacity, and bodily wholeness [3, 7, 9]. The Dual Process Model illuminates how mastectomy tattoos may facilitate both coping processes simultaneously: They provide opportunities to confront and process loss while also serving as restoration-oriented activities that help reconstruct identity and create new meaning.

Complementing this framework, Neimeyer’s [21, 22] meaning reconstruction theory emphasizes that grieving involves constant reconstruction of meaningful understanding. As Neimeyer [21] articulates: “Like a novel that loses a central character in the middle chapters, the life story disrupted by loss must be reorganized, rewritten, to find a new strand of continuity that bridges the past with the future in an intelligible fashion” (p. 263). Mastectomy tattoos may serve precisely this function: providing a medium through which women can rewrite their bodily narratives, making sense of traumatic experiences and restoring life narrative coherence.

### Research gap and study rationale

A growing body of literature has begun to document women’s experiences with post-mastectomy tattooing specifically. Using interpretative phenomenological analysis with eight women, Newlan and Greig [23] described a trajectory from bodily disruption to a “second marking” of the body through tattooing, involving transformation, acceptance, and renewed control. Dann and Callaghan [24] similarly highlighted the collaborative process between tattoo artists and clients in constructing personally meaningful body narratives, while Tsang [25] explored tattooing more broadly as a form of personal storytelling and coping. From a clinical perspective, Allen [26] described how decorative mastectomy tattoos can help women transform feelings of disfigurement into a renewed sense of beauty and self-determination.

Despite growing interest in mastectomy tattoos among patients and media attention to this practice, empirical research examining their psychological significance remains limited. While existing studies have documented positive effects on body image [16] and identified tattoos as forms of identity construction [18], few have systematically examined how women experience these tattoos as coping mechanisms or explored the meaning-making processes they facilitate. Moreover, little research has employed visual analysis methods to understand the symbolic content of mastectomy tattoos themselves, missing an opportunity to examine how visual elements communicate meaning and support psychological recovery.

Given the minimally invasive nature of post-mastectomy tattoos, whose risks are generally limited to localized skin reactions such as infection, allergic response, and scarring, with severe complications reported as rare [27], combined with their demonstrated physical, emotional, and psychological benefits, there is a compelling need to better understand their therapeutic potential. Such understanding could inform clinical practice, helping healthcare providers incorporate discussions of artistic tattooing into post-surgical planning. Additionally, evidence supporting the psychological benefits of mastectomy tattoos could strengthen advocacy for insurance coverage, moving toward funding parity with other post-mastectomy cosmetic procedures [10].

### The present study

This study explores how women utilize post-mastectomy tattoos as coping mechanisms, examining their impact and associated meaning-making processes. Through qualitative analysis combining semistructured interviews with social semiotic analysis of tattoo imagery, we investigate the symbolic and narrative meanings women attribute to these artistic expressions. Specifically, we explore how women describe and make sense of the role mastectomy tattoos play in their relationship with their bodies, their sense of self, and their experience of embodiment following bodily loss. By integrating women’s lived experiences with analysis of the tattoos themselves, this research aims to broaden understanding of coping mechanisms available to women undergoing mastectomy and highlight the potential role of artistic tattooing as an additional supportive resource within comprehensive post-mastectomy care. Potential pathways for integrating this practice into clinical settings, including interdisciplinary referral processes, are discussed further in the “Clinical implications and conclusions” section.

## Methods

This study employed a qualitative research design, combining two data sources: semistructured interviews and photographs of participants’ tattoos—analyzed respectively through reflexive thematic analysis and social semiotic analysis, to explore the experiences of 12 women who had undergone mastectomy and subsequently chosen to get mastectomy tattoos. Ethics approval was obtained from the Institutional Review Board (IRB) of The Academic College of Tel Aviv-Yaffo (protocol number 66) in accordance with the Declaration of Helsinki.

This study was situated within a contextualist epistemological framework [28], which occupies a middle ground between realist and constructionist positions. This stance recognizes participants’ accounts as reflecting meaningful, genuine experiences of their bodies and lives, while also acknowledging that meaning is actively and collaboratively constructed by participants and researchers throughout the research process. This position informed our use of thematic analysis to identify patterns of meaning across interviews, while remaining attentive, through reflexive practice and peer debriefing, to the interpretive nature of that analytic process.

### Participants and recruitment

Participants comprised 12 women who underwent mastectomy following breast cancer treatment and subsequently chose to get a post-mastectomy tattoo. Participants were identified by pseudonyms: Dalia, Ariel, Miriam, Shira, Michaela, Abigail, Hannah, Maya, Jordan, Neomi, Rachel, and Eden. Ages ranged from 30 to 56 years (*M* = 44.5, SD = 8.3).

Sample size was determined based on principles of qualitative research saturation. Guest et al. [29] found that saturation often occurred within the first 12 interviews, although basic elements of meta-themes were present as early as six interviews. Saturation in qualitative research signifies that further interviews or data collection efforts are unlikely to reveal new insights, themes, or patterns relevant to the research questions. Based on these principles and the richness of data emerging during analysis, 12 participants provided sufficient depth for meaningful thematic development. Demographic information collected included age, employment, city of residence, age at treatment completion, and age when diagnosed with cancer. Participants were recruited through purposive sampling via online breast cancer forums, tattoo-related social media groups, and tattoo studios specializing in post-mastectomy tattooing. Recruitment materials were disseminated exclusively in Hebrew, and interviews were conducted in Hebrew; participation was therefore limited in practice to Hebrew-speaking women, accounting for the linguistic homogeneity of the sample. Women who expressed interest in participating received further information regarding the study and provided informed consent prior to participation.

Recruitment was deliberately focused on women who had chosen decorative artistic tattooing, rather than NAC tattooing, in order to align with the study’s aim of exploring tattooing as a form of symbolic meaning-making and identity reconstruction, as distinct from anatomical reconstruction. Women who had undergone NAC tattooing exclusively, without a decorative component, were not included in the present sample.

The choice to exclusively recruit women stemmed from their predominant representation in breast cancer diagnoses [30]. While recognizing the importance of research with women, we acknowledge that breast cancer and post-mastectomy tattoos extend beyond individuals identifying as women. Participant demographic and tattoo characteristics are presented in Table 1.

**Table 1:** Participant demographic and tattoo characteristics. Participants ranged in age from 30 to 56 years (mean = 44.5, SD = 8.3). All participants underwent either mastectomy or lumpectomy followed by artistic tattoo application. Tattoo designs varied widely, incorporating natural imagery (flowers, butterflies, birds) and abstract patterns reflecting themes of transformation, freedom, and connection to life

	Dalia	Ariel	Miriam	Shira	Michaela	Abigail	Hannah	Maya	Jordan	Neomi	Rachel	Eden
Age	56	33	51	50	30	41	38	54	44	48	47	42
M/L^a^	L	M	M	M	M	L	M	M	M	M	M	M
Tattoo design	Almond tree branch, flowers	Lotus flower, color	Flowers, butterflies, leaves, colorful	Geometric body flow, abstract	Geometric figures, abstract	Flowers, leaves		Flower, abstract	Bird, fruit, flowers, nest, eggs, colorful	Butterflies, leaves	Flowers, sunflower, leaves, butterflies, colorful	Flowers, leaves

^a^Mastectomy/lumpectomy

### Data collection

Data were collected through semistructured interviews with women who had undergone a single or double mastectomy and subsequently chose to get mastectomy tattoos. Interviews were conducted between June 2023 and April 2024 via the Zoom platform.

We opted for semistructured interviews due to their inherent openness, which provided flexibility and facilitated in-depth conversations while maintaining consistency across participants. While asking predetermined questions in a fixed sequence facilitates easy comparison among respondents, it can also impose constraints. In contrast, embracing a more fluid structure can unveil underlying patterns [31].

The interview guide included open-ended questions designed to elicit detailed narratives about participants’ experiences with mastectomy, their decision to get tattoos, and the meanings they attributed to their tattoos. One example question from the interview was “Did mastectomy tattoos affect your relationship with your body and your breasts? In what ways?” Additional questions explored bodily perceptions post-surgery, the tattoo design process, and experiences of living with mastectomy tattoos.

The interviews were video and audio recorded and lasted approximately 45 min (range 35–60 min). All interviews were conducted in Hebrew, the participants’ native language, and subsequently transcribed verbatim. Data analysis, including coding and theme development, was conducted on the original Hebrew transcripts to preserve linguistic and cultural nuance. Only the quotations selected for inclusion in the manuscript were translated into English, using direct translation with an emphasis on conceptual rather than strictly literal equivalence, refined to read naturally in English while preserving participants’ intended meaning. In addition to interview data, participants were invited to share photographs of their tattoos, which were analyzed using social semiotic methods (described in the “Social semiotic analysis” section). Participants provided two separate and independent consent forms: one for participation in the interview and a second specifically for the sharing of tattoo photographs. Submission of a photograph was therefore optional and not required for participation in the study. Of the 12 interviewed participants, 11 chose to share at least one photograph of their tattoo; four of these participants shared multiple images taken from different angles. Representative examples of participants’ mastectomy tattoos are provided as supplementary material.

### Data analysis

Data analysis employed two complementary analytical approaches: thematic analysis of interview transcripts and social semiotic analysis of tattoo images. This multimethod approach allowed for triangulation of findings and deeper understanding of both the lived experiences and visual symbolism of mastectomy tattoos.

#### Thematic analysis

Interview transcripts were analyzed using thematic analysis to identify patterns of meaning across participants’ narratives. The analysis followed an inductive approach, with codes and themes generated directly from the data rather than being derived from a pre-existing coding framework; the Dual Process Model [19] was subsequently applied during “Discussion” to interpret and contextualize the emergent themes. Braun and Clarke’s [28] six-phase model was followed. The first phase, familiarization with data, involved transcribing all 12 interviews and reading them multiple times to familiarize the researchers with the material. In the second phase, generating initial codes, codes were generated inductively within individual transcripts to capture specific, meaning-based features of the data (e.g., a participant describing avoidance of mirrors or associating a tattoo symbol with liberation from illness). Codes did not need to be present across all transcripts to be retained; rather, each code reflected a discrete unit of meaning grounded in the data. In the third phase, searching for themes, codes were collated into potential themes, including “sense of control,” “belonging,” and “focusing on restoration plans.” During the fourth phase, reviewing themes, these themes were refined and organized. For example, preliminary codes related to transformation and growth were consolidated into the broader theme “mastectomy tattoos as reclaimed identity.” In the fifth phase, defining and naming themes, each theme was clearly defined and named. The final phase, producing the report, involved selecting compelling examples that were analyzed and discussed in relation to the study aims and existing literature [28].

#### Social semiotic analysis

Social semiotics examines how meaning is created and communicated through visual elements [32, 33]. This approach recognizes tattoos as powerful forms of sign-making, where both tattoos and clients construct meaning through symbols and visuals.

Following Kress and Van Leeuwen’s [33] framework for visual analysis, we examined compositional elements of tattoo images including *information value*: placement of elements (left, right, top, bottom, center, margins) and their associated meanings; *salience*: techniques such as size, color, and contrast that draw attention to particular elements; *framing*: visual boundaries or arrangements that connect or separate elements within the tattoo.

Analysis proceeded in two stages: First, we identified visual components of each tattoo (e.g., flowers, butterflies, birds, abstract patterns) and documented their compositional arrangement. We asked: How are elements arranged? How do components interact visually? What elements are most salient? How does placement on the body (e.g., covering mastectomy scars) influence meaning? Where relevant, participants also addressed the practical considerations underlying placement decisions during their interviews—for example, choosing a design specifically to cover a scar, or conversely, positioning an element deliberately to mark and commemorate rather than conceal a particular site on the body. These considerations, when raised by participants, were incorporated into the interpretation of compositional meaning.

Second, after analyzing each tattoo individually using this framework, we identified recurring symbols and themes across participants’ tattoos. These theory-driven interpretations were then cross-checked against each participant’s own narrative account for consistency with her individual story. Where a participant explicitly explained the meaning of a specific visual element during her interview, this explanation was used to ground the interpretation directly in her own words. Where no such explicit explanation was available, the interpretation is presented as a plausible symbolic reading within the social semiotic framework, rather than as a claim about the participant’s stated intention. This integration of visual and verbal data allowed for richer interpretation of how mastectomy tattoos function as coping mechanisms and meaning-making tools [18, 34].

### Ensuring rigor and trustworthiness

The research team approached this study with awareness of the significant trauma and loss experienced by women following mastectomy. While recognizing that we cannot fully comprehend the depth of their experience, we sought to understand how mastectomy tattoos function as coping mechanisms and means for women to regain a sense of control through body art. Given the challenges these resilient women have faced, the research team approached this research with deep empathy and respect to better understand their journeys.

To ensure trustworthiness and credibility of findings, several strategies were employed. Peer debriefing was conducted throughout the analytical process, with colleagues and doctoral students independently reviewing interview transcripts and emerging themes, offering valuable insights and alternative perspectives. This collaborative review process enhanced comprehensiveness and reliability of the thematic framework [35]. Additionally, reflexive practice was maintained throughout data collection and analysis, with regular discussions among the research team about emerging interpretations and potential biases.

## Findings

**Fig. 1:**
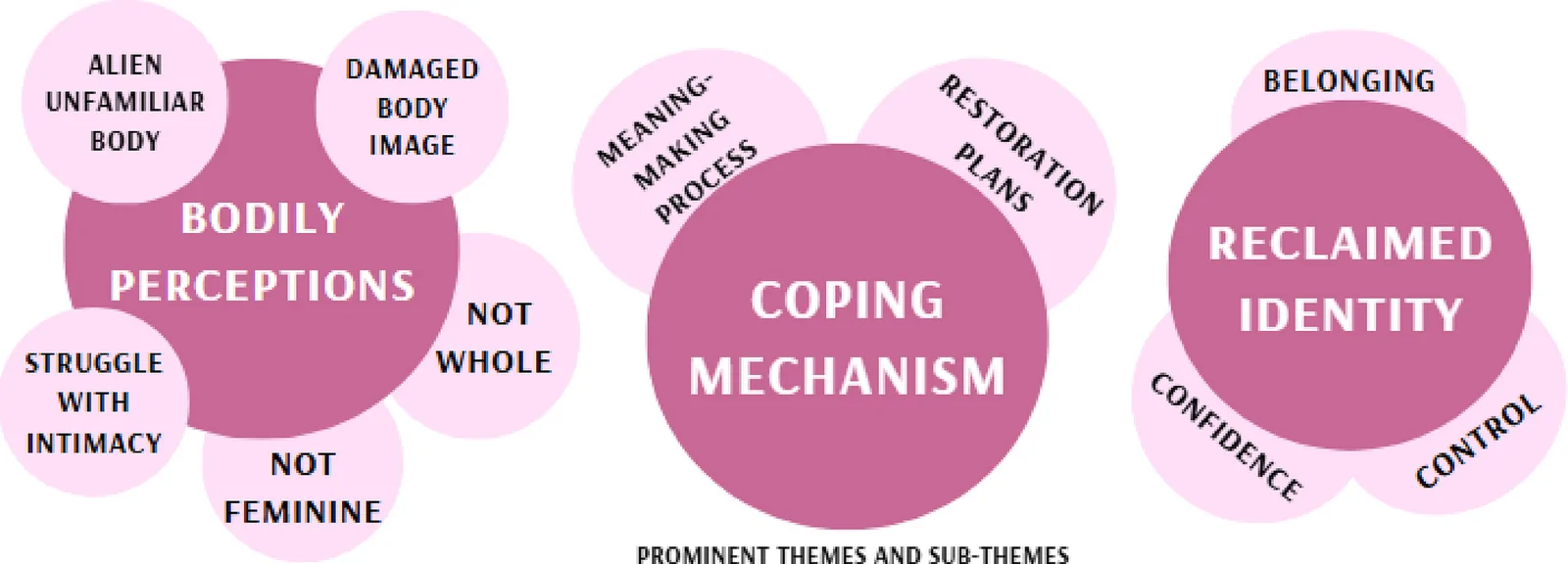
Thematic structure of findings. Visual representation of the three primary themes and their subthemes identified through thematic analysis of interview transcripts. Theme 1 (bodily perceptions post-surgery) encompasses five subthemes describing post-surgical experiences. Theme 2 (mastectomy tattoos as a coping mechanism) includes two subthemes characterizing the therapeutic planning process. Theme 3 (mastectomy tattoos as reclaimed identity a constant reminder) contains three subthemes reflecting ongoing psychological benefits

Analysis of interview transcripts and tattoo images yielded complementary findings that illuminate the psychological significance of mastectomy tattoos. Thematic analysis of interviews identified three primary themes describing women’s experiences before and after receiving mastectomy tattoos. The thematic structure is illustrated in Fig. 1. Visual semiotic analysis of tattoo imagery revealed recurring symbolic patterns that reflected participants’ meaning-making processes. Together, these findings demonstrate how mastectomy tattoos function as both coping mechanisms and tools for identity reconstruction.

### Thematic analysis

#### Theme 1: bodily perceptions post-surgery

The prospect of mastectomy represents a profound alteration to one’s body and identity. Many women found themselves confronting a new body image that felt unattractive, unfeminine, unfamiliar, or disjointed from their presurgery selves. Eden’s reflection following breast cancer diagnosis and mastectomy captures this painful realization.

All of a sudden, I comprehended the meaning of this whole thing. I came out of the room crying, like I haven’t cried all this time. Because I said “What! I won’t have breasts, I won’t have a nipple. What!”. It’s like a boom. A real boom. [Eden]

Eden’s distress reveals the emotional intensity of confronting bodily loss. For Eden, the realization that she would “not have breasts, [or] a nipple” marked a sudden and overwhelming confrontation with the reality of her surgery—what she described as “a real boom.” This moment of recognition illustrates the intensity with which the loss registered emotionally, though participants’ accounts point to multiple, sometimes overlapping dimensions of that loss—bodily, emotional, and relational—rather than a single, uniform disruption of “self.” Breasts and nipples are not only physical attributes but are deeply tied to personal identity and self-image. Miriam’s statement reflects the traumatic loss of her familiar feminine form:

After going through such a trauma of a mastectomy, a breast is a very feminine thing. It’s not a reduction, and it’s not an enlargement, it’s something completely different. It’s a breast removal. You lose the breast, you lose the nipple, you lose the shape. [Miriam]

Miriam’s repetition of “you lose” emphasizes the totality of what was lost. The removal of breasts and nipples evoked disconnection from one’s body, leading to grief over loss of wholeness, belonging, and femininity. Following mastectomy, all participants described profound disruptions to their sense of embodiment. Five interrelated subthemes characterize these post-surgical experiences.

- Subtheme 1.1: damaged body image

The surgical removal of breasts and nipples resulted in severe body image disturbance for all participants. Women described intense aversion to their post-surgical bodies and engaged in avoidance behaviors to minimize confrontation with their altered appearance. Neomi’s account, describing her experience prior to receiving her tattoo, reveals the depth of this distress:

I would take a shower for maybe 3 seconds. I swear, like, even when I would take a shower or get dressed, since then I couldn’t look at myself in the mirror, I would never look at myself in the mirror at all. I couldn’t bear the fact that I don’t have a nipple, and the breast deformed. [Neomi]

For some participants this distress has lasted years. Eden described 3 years of deliberately avoiding her reflection:

For three years I didn’t look at myself in the mirror after a shower. I showered, got dressed, and that’s it... It’s a scar from end to end. Imagine, on both breasts. I don’t remember what my nipples used to look like. [Eden]

- Subtheme 1.2: an alien unfamiliar body

Beyond damaged body image, participants described their post-surgical body as unreal, foreign, and disconnected. This alienation was compounded by loss of sensation in reconstructed breast tissue. Abigail described the constant awareness of bodily asymmetry and deformity:

Apart from scars, asymmetry remained, and I had deformity… It’s constantly noticeable, especially when I’m lying on my back- you can see a big dent. I couldn’t look at myself in the mirror. [Abigail]

Hannah described loss of physical sensation:

For many years, that it was not mine. With any feeling gone, you feel like you carry two stones, two rocks on you. Both the physical feeling and appearance feel foreign, as if they’re not part of you. [Hannah]

- Subtheme 1.3: feeling not feminine

Participants explicitly linked breast removal to loss of femininity. Shira’s description of her post-surgical body as feeling like “an elbow” reflected this experience: “It looked weird and didn’t look like a breast …” [Shira]. Ariel’s statement also referenced breastfeeding: “This breast cannot breastfeed. And it’s not a breast either. It is not real…” [Ariel].

Eden’s perspective further illustrates the connection between breasts and femininity: “It lowers femininity. The breast was never sexy in my eyes, because I nursed, and it was baby food. Not something really feminine and sexy, but it’s still a female organ” [Eden].

- Subtheme 1.4: feeling not whole

Participants described feeling fragmented and incomplete following mastectomy. Jordan’s statement described both the physical absence and emotional void:

It was very difficult to see it. Not only the scar but what’s missing. The hole. Where there are no breasts, there should be breasts... I had 3 years to deal with the fact that they were for beauty. They didn’t have much role... [Jordan]

Jordan’s reflection illustrates the persistence of this sense of absence even when it is reasoned through: Although she came to regard her breasts as having served a primarily aesthetic rather than functional role in her life, their physical absence still registered as a void that could not be resolved by that reasoning alone.

- Subtheme 1.5: struggling with intimacy

Subtheme 1.5: struggling with intimacy

The physical and psychological changes following mastectomy affected intimate relationships. Women reported self-consciousness about scars and avoidance of intimate situations. Neomi described her experience: “It was very difficult for me. I couldn’t, I wouldn’t look at myself in the mirror. No way, no way. Sex with my partner…only with a tank top. No way, no way” [Neomi]. Eden’s experience reflected similar concerns; as she had already described avoiding mirrors for 3 years, this extended to her intimate relationship as well: “…and also the relationship with my husband, I wouldn’t take off my shirt. It lowers your confidence…” [Eden].

These experiences of bodily disruption, alienation, and loss characterized all participants’ immediate post-surgical period.

#### Theme 2: mastectomy tattoos as a coping mechanism

The decision to get mastectomy tattoos marked a turning point in participants’ post-surgical journeys. Tattoos served multiple coping functions, both during the planning process and after completion. Two subthemes describe how tattoos facilitated psychological adjustment.

- Subtheme 2.1: focusing on restoration plans

The process of planning and designing mastectomy tattoos provided participants with a future-oriented project. Maya’s extended deliberation illustrates this process:

I deliberated for a long time, considering that maybe I could fix it and it would look nice. You know, it’s hard to give up the possibility that it might actually turn out beautiful. Then, at some point, I made a decision… even if I fixed it and it looked good, I would still get some kind of tattoo…. [Maya]

Dalia described similar extended planning:

So she drew it for me and sent me the sketch, which I kept on my phone for about two years. The idea was to create a tattoo that would cover, on one hand, that annoying spot that bothered me: the additional mark on the place where the tumor had supposedly been... I was looking at the sketch as I had it saved in a folder somewhere, and I didn’t really know what to do with it or if I was actually going to go through with it. [Dalia]

Jordan’s planning began even during active treatment:

Even during chemotherapy, I said to myself that as soon as I could, I was going to get a tattoo on that spot... And indeed, it was something that occupied my mind, what I would tattoo, what it would be, and I had a lot of time to think about it. The tattoo was done a year after I finished radiation therapy… I went through a process of choosing what to get and how to choose it, and I really spent a lot of time on it. It was something that occupied me a lot, what I was going to put there. [Jordan]

- Subtheme 2.2: meaning-making process

Participants selected tattoo designs that held personal symbolic meaning. Miriam explained her choice of a butterfly:

Why did I place a butterfly in the center? A butterfly is a happy creature; it appears for a short time but is always free. For me, the liberation from the breast was like becoming a butterfly, gaining freedom. To be free from the enemy that was attached to my body. [Miriam]

The extended planning process and symbolic design choices reflected participants’ active engagement in reconstructing meaning following mastectomy.

#### Theme 3: mastectomy tattoos as reclaimed identity

Following tattoo completion, participants described profound and lasting changes in their relationships with their bodies. Three subthemes characterize these ongoing experiences.

- Subtheme 3.1: sense of belonging

Tattoos helped participants reclaim bodies that had felt alien following surgery. Abigail described this experience:

This is the first time I felt “they’re mine.” As if I never had a sense of belonging to the new breasts and after he did the tattoo I said “They’re mine.” It literally looks like it’s my chest. It’s very touching. [Abigail]

Rachel also described ongoing connection to her tattooed body: “I also really like them a lot, I also like to look at it [the tattoo] myself. Let’s say if I get out of the shower, I always look at it. It’s already a part of me” [Rachel].

- Subtheme 3.2: sense of confidence

Tattoos restored confidence that had been damaged by mastectomy. Participants reported increased comfort with bodily exposure. Miriam described this shift:

It’s a gift to do it [the tattoo] to yourself. It really makes the heart happy. It’s happy, it also gives back some kind of confidence. I can tell you that for a long time I didn’t go into the women’s locker rooms and take a shower, because it drew attention. Today when it attracts eyes, I even like it. I also undress in front of myself with affection many times. And I think if it makes me feel good, that’s what matters. [Miriam]

Shira described similar restoration of confidence manifested in clothing choices:

It did give me a lot of confidence, and I love it and everyone asks me. Let’s say I put on a swimsuit. I started wearing a bikini after thirty years of not wearing a bikini, I think, since I was 20. [Shira]

- Subtheme 3.3: sense of control

Participants described tattooing as reclaiming personal autonomy after cancer. Michaela explicitly contrasted tattoo choice with medical necessity*:*

I did the tattoo to feel control over my body. All the medical stuff I had to do that year before, wasn’t really by choice. Getting the tattoo was like: Okay, this is what I really choose to do to my body. It is a choice that you know you will always have to be whole with... I can decide when I want to show it and when I don’t want to show it... That was the last note I chose to play… [Michaela]

Miriam described her design choices:

I drew exactly what I thought of drawing, I drew butterflies, I wrote on the left shoulder “freedom” because I’m free from the subject “breast” and on the right side I wrote “health.” I mean, my tattoo speaks volumes. On the one hand is freedom, on the other hand is health… [Miriam]

Participants described their tattoos as fostering renewed sense of belonging to their bodies, increased confidence in social situations, and restored personal control following a period of medical necessity.

Together, these three themes illustrate participants’ journeys from profound post-surgical distress, through active engagement in tattoo planning and design, to restored sense of bodily belonging, confidence, and personal control.

### Visual analysis findings

Social semiotic analysis of participants’ mastectomy tattoos revealed a set of recurring visual and symbolic patterns, expressed through natural imagery, compositional choices, and figurative elements. Representative examples of mastectomy tattoos illustrating these patterns are provided in Supplementary Material.

Across participants’ tattoos, natural imagery—flowers, vines, branches, and fruit—featured prominently as a visual language for growth and transformation. Open flowers symbolized blooming and thriving, while closed flowers indicated ongoing emotional recovery or potential yet to unfold. Dalia’s almond tree branch, with its delicate blossoms, reflected hope and renewal, while Ariel’s lotus flower, symbolically associated with spiritual transformation, highlighted strength and beauty emerging from hardship.

Closely related to this imagery of growth was a recurring emphasis on freedom, conveyed particularly through butterflies, birds, and abstract, fluid forms. Jordan’s, Miriam’s, and Neomi’s butterfly and bird tattoos exemplified this sense of liberation from physical and emotional constraints, while Shira’s abstract design, with its flowing lines, suggested movement rather than stasis.

These images of growth and freedom were, in turn, embedded within a broader visual vocabulary of connection to nature and life: nests, eggs, leaves, and branches recurred alongside the floral and animal imagery already described. Jordan explained that her nest held three eggs representing her three children, each linked to a bird reflecting that child’s personality: “They were really part of this… so they are all present within this tattoo” [Jordan]. This account grounds the nurturing symbolism directly in the participant’s own explanation. Dalia’s branch similarly symbolized a firm connection to life. Shira’s abstract leaves and arrows suggested openness to new experiences. Together, these interwoven visual elements—natural forms, figures of flight, and symbols of nurturing and connection—reflected shared processes of growth, liberation, and vitality expressed through participants’ tattoos.

Taken together, these visual patterns extend and reinforce the narrative themes identified through the interviews. Rather than functioning solely as aesthetic modifications, the recurring imagery of blooming, flight, and connection to nature suggests that tattoos served participants as external, visible representations of an internal process of meaning-making and identity reconstruction. Within the framework of the Dual Process Model, these visual choices can be understood as restoration-oriented: Through symbols of growth, freedom, and vitality, participants visually reclaimed ownership over bodies that had, following mastectomy, felt alien or diminished. The convergence between women’s verbal accounts and the symbolic content of their tattoos points to a coherent process in which loss is not erased but actively reframed and integrated into a renewed sense of self.

## Discussion

This study explored how women utilize post-mastectomy tattoos as coping mechanisms and meaning-making processes following mastectomy. Analysis revealed three interrelated narrative themes describing participants’ trajectories from profound post-surgical distress (theme 1: bodily perceptions post-surgery), through active engagement in tattoo planning (theme 2: mastectomy tattoos as a coping mechanism), to restore sense of embodiment (theme 3: mastectomy tattoos as reclaimed identity). Visual semiotic analysis complemented these narrative findings, revealing symbolic patterns of personal growth and transformation, freedom, and reconnection to nature and life. Together, these findings illuminate how mastectomy tattoos serve dual functions within the grief process: facilitating confrontation with loss while enabling restoration-oriented coping and identity reconstruction.

For many women with breast cancer, tattoos help shape their identity in relation to their illness, symbolizing resilience and strength against physical and social consequences of cancer [18]. Participants in this study described their tattoos as symbols of freedom from cancer, victory, renewal, and ongoing reminders of their strength. Some viewed their tattoos as means of taking control of their bodies and asserting agency in telling their stories. Tattoos serve as personal storytelling, transforming traumatic experiences of breast cancer into narratives of strength and survival [8, 36].

These findings align with previous research by Tsang [25], suggesting that tattoos have therapeutic benefits by allowing individuals to cope with significant life challenges and symbolize their triumphs. This is consistent with broader findings on medical and cosmetic tattooing: A systematic review of patient satisfaction across diverse clinical applications reported consistently high satisfaction outcomes [37], and a recent large-scale survey found that breast cancer survivors with medical tattoos reported significantly lower body image distress and psychological symptoms compared to those without [38]. Mastectomy tattoos can help women alter perceptions of their bodies, regain control, and foster positive changes in their interactions with the world [17]. The current study extends this literature by documenting specific ways tattoos facilitate both loss-oriented and restoration-oriented coping processes, demonstrating how the extended planning period serves therapeutic functions even before tattoo completion.

### Loss-oriented coping

Loss-oriented coping involves directly confronting and processing emotional and psychological impacts of loss [20]. Breast removal evokes profound grief because breasts are often central to self-image, sexuality, and femininity. Mourning responds to not only physical loss of breasts but also the loss of identity and sense of wholeness. Theme 1 (bodily perceptions post-surgery) vividly illustrated this grief, with all participants describing profound disruptions to their sense of embodiment. Participants described feelings of alienation and discomfort with their bodies and struggles with intimacy and body image [4, 23, 26]. Neomi’s 3-s showers and Eden’s 3-year avoidance of mirrors exemplify the intensity of post-surgical body image disturbance. Hannah’s metaphor of carrying “two stones, two rocks” powerfully conveys the corporeal alienation-reconstructed breasts felt like external objects rather than integrated parts of the self. This distress was not resolved during the healing period itself; as detailed in the following section, participants’ movement toward reconciliation with their bodily changes was closely tied to the process of designing and receiving their tattoos.

Herman [15] notes that traumatic events like mastectomy lead to detachment from identity, when breasts become symbols of loss and disruption. In this study, several women (including Abigail, Neomi, and Eden) shared how they avoided looking at themselves in mirrors and felt disconnected from their reflections, experiencing ongoing difficulty coming to terms with their new body images. The loss extended beyond physical attributes to encompass femininity and maternal identity. Ariel’s statement that her reconstructed breast “cannot breastfeed” and “is not real” illustrates how mastectomy disrupted multiple interconnected aspects of female identity. Even Eden, who had not experienced her breasts as “sexy,” recognized their removal as loss of a “female organ,” indicating that femininity encompasses multiple, sometimes contradictory meanings.

### Restoration-oriented coping

Restoration-oriented coping focuses on adapting to new reality and finding ways to rebuild and make meaning of traumatic experiences [20]. Mastectomy tattoos play crucial roles in this coping process by allowing women to reclaim their identity, confidence, and belonging [8, 24]. By choosing meaningful and personalized designs, women shift focus from loss to renewal [21]. Theme 2 (mastectomy tattoos as a coping mechanism) illustrated how this restoration-oriented work began even before tattoo completion.

The extended planning process itself appeared to serve important psychological functions for participants. Maya’s extended deliberation, oscillating between action and inaction for months, illustrates how planning offered a space to sit with the decision before committing to a permanent change. The possibility that her tattoo “might actually turn out beautiful” represented hope and a way forward that did not require denying loss. Dalia kept a tattoo sketch on her phone for 2 years, allowing her to “try on” the idea without commitment. Jordan’s planning began during chemotherapy itself, providing a sense of control during a period of medical interventions over which she had little agency. The tattoo represented something she could choose—a deliberate act of self-determination contrasting with medically necessary procedures. Mastectomy tattoos are not merely aesthetic choices; they represent acts of restoration. Dann and Callaghan [24] and Tsang [25] suggest that collaborative processes between tattoo artists and individuals are essential in capturing personal stories and creating tattoos that reflect unique experiences. Participants carefully selected symbols that held deep personal significance. Miriam’s butterfly represented multiple meanings simultaneously: freedom, happiness, transformation, and liberation from cancer (“the enemy”). By framing breast removal as “liberation,” Miriam recast loss as gain—freedom from disease. This cognitive reframing, externalized through the tattoo, provided an alternative narrative to one of pure loss.

Mastectomy tattoos represent responses to physical and emotional fragmentation experienced after breast removal, offering women opportunities to “correct” perceived deficits and enhance their sense of wholeness. Tattoos facilitate psychological healing, reconstituting body and identity by transforming scars into symbols of empowerment [12, 15, 18]. Theme 3 (mastectomy tattoos as reclaimed identity) demonstrated the lasting impact of this restoration. Abigail’s statement: “This is the first time I felt ‘they’re mine’,” captured a transformative moment of recognition and ownership. The tattoo marked the point at which her chest ceased feeling like foreign objects and became integrated into her embodied self. In stark contrast to post-surgical mirror avoidance, Rachel now regularly and pleasurably gazed at her tattooed chest: “I also like to look at it myself. Let’s say if I get out of the shower, I always look at it.”

Although participants typically pursued tattooing after the initial period of physical healing, the psychological process was not linear. Deciding to undergo tattooing required renewed engagement with the mastectomy scar, bodily change, and unresolved grief, while simultaneously enabling participants to reconstruct their identity and restore a sense of agency. Rather than representing a purely restoration-oriented intervention, tattooing therefore appears to constitute an embodied process through which women repeatedly moved between confronting the emotional consequences of breast loss and reconstructing a renewed sense of self—illustrating the oscillation between loss- and restoration-oriented coping that is central to the Dual Process Model [19, 20].

Our findings thus suggest that tattooing represents an active coping practice through which women engage simultaneously with loss, embodiment, identity reconstruction, and restoration, rather than a passive outcome of psychological adjustment.

### Reclaiming identity through tattooing

Participants’ accounts suggest that tattoos may function as a means of externalizing internal pain, transforming it into a narrative of personal meaning and strength—for example, through Michaela’s description of the tattoo as “the last note I chose to play” or Miriam’s incorporation of the words “freedom” and “health” directly into her design. This process allows women to reconstruct their identities by transforming their bodies into sites of empowerment and personal significance [13]. By integrating meaningful symbols, individuals reclaim control over their bodies, reasserting their identity and counteracting negative effects of breast removal. Thus, tattoos are not merely decorative but represent a deeper, psychological restoration of self.

The restoration of confidence was particularly striking. Miriam’s transformation from avoiding women’s locker rooms to welcoming attention illustrates how tattoos shifted the meaning of bodily visibility. Rather than exposure revealing perceived deficiency (missing breasts), it now showcased artistry and personal expression. As shown in theme 3, this shift from shame to pride was echoed in Shira’s return to wearing bikinis after 30 years. Critically, participants also described tattooing as reclaiming bodily autonomy after a period of medically mandated interventions: Michaela’s contrast between medical obligation and self-determined choice, discussed above, exemplifies this shift from passive patienthood to active agency. Similarly, Miriam designed her own tattoo and incorporated text (“freedom,” “health”), literally inscribing her interpretation of her experience onto her body. The tattoo “speaks”—tells a story—but it is her story, authored by her rather than imposed upon her.

Visual analysis complemented these narrative accounts, revealing how symbolic content communicated transformation, liberation, and renewed connection to life. Natural imagery—flowers, branches, nests—predominated, suggesting participants conceptualized their post-cancer journeys as organic processes of growth. Open flowers symbolized thriving despite trauma, while branches represented connections to larger life systems. Butterflies and birds emphasized freedom and transcendence. The visual patterns aligned with verbal narratives, demonstrating consistency across modalities in how women made meaning of their experiences.

### Limitations

This study has several limitations. One challenge involved translating qualitative data from Hebrew to English, which may have resulted in the loss or alteration of cultural nuances and emotional connotations inherent in participants’ experiences with mastectomy tattoos. Future research should involve bilingual researchers sensitive to cultural contexts and incorporate member checking to ensure accurate representation of participants’ experiences [39].

Additionally, all 12 participants had chosen to undergo artistic mastectomy tattoos and were willing to participate in research; the sample therefore does not include women who considered but ultimately decided against tattooing. This self-selection may mean participants had particularly positive experiences with tattooing, potentially limiting transferability of findings to all women who undergo mastectomy. Women who chose not to get tattoos, or who had negative experiences with tattoos, are not represented in this study.

The study was conducted in a specific cultural context, which may influence both the meaning of mastectomy and the acceptance of body modification practices. Cultural attitudes toward tattoos, femininity, and breast cancer vary across societies, potentially limiting generalizability of findings to other cultural settings. This cultural specificity, combined with the self-selected nature of the sample, may also have influenced the point at which thematic saturation was reached.

Finally, this study employed a cross-sectional design, interviewing participants at a single time point after tattoo completion. A longitudinal approach examining women’s experiences before, during, and after the tattooing process could provide richer understanding of how meaning-making evolves over time. Future research might also explore whether the psychological benefits of mastectomy tattoos are sustained long-term or whether they shift as women continue to adjust to life after breast cancer.

### Clinical implications and conclusions

This study demonstrates that mastectomy tattoos serve as multifaceted coping mechanisms that address both loss-oriented and restoration-oriented processes within the Dual Process Model [19, 20]. These tattoos play critical roles in helping individuals confront and process grief while facilitating reconstruction of identity and meaning. Visual analysis revealed symbolic patterns of growth, freedom, and renewed connection to life that complemented participants’ narrative accounts. By addressing the bodily alienation and fragmentation often experienced after surgery, mastectomy tattoos provide opportunities for meaning-making and identity reconstruction. This is achieved by creating new external symbols that absorb new meanings and reflect life-changing events on the body.

Mastectomy tattoos act as a means of reclaiming identity after cancer and serve as bridges, linking women’s internal experiences with their renewed sense of self and external appearance. This process helps women become confident, integrated, and whole again [12]. Critically, the planning process itself serves therapeutic functions, offering future-oriented focus and sense of control during a period when women may feel their bodies have been subject to medical necessity rather than personal choice.

Clinicians, especially medical and rehabilitation psychologists working with cancer patients, should be aware of the coping and meaning-making processes women experience following mastectomy. Healthcare providers should inform patients about all available options, including artistic mastectomy tattoo, and their potential therapeutic benefits.

Given the range of professionals involved in post-mastectomy care—including oncologists, breast and plastic surgeons, oncology nurses, psychologists, and social workers—interdisciplinary collaboration is essential to ensure women receive comprehensive and balanced information about all reconstructive and aesthetic options. Establishing clear referral pathways to qualified medical tattoo artists, alongside existing referral processes for reconstructive surgery, would help ensure that women who express interest in post-mastectomy tattooing can access this option as part of coordinated, multidisciplinary care.

Given the minimally invasive nature and low risk profile of post-mastectomy tattoos, combined with their demonstrated psychological benefits, healthcare systems should consider incorporating artistic tattooing into funded post-mastectomy care options, moving toward funding parity with other cosmetic procedures such as breast reconstruction and medical nipple tattoos.

Future research should continue exploring the therapeutic mechanisms underlying mastectomy tattoos, with attention to making them more accessible and available to diverse populations. Longitudinal studies examining the durability of psychological benefits over time would strengthen the evidence base for clinical recommendations. Additionally, research comparing outcomes between women who choose tattoos, reconstruction, or no intervention could inform personalized treatment planning. This evidence base would support women who have had their breasts removed in finding pathways to “feel like they are theirs again.”

## Supplementary Information

Below is the link to the electronic supplementary material.

ESM 1 (PDF 265 KB)

## Data Availability

The qualitative data (interview transcripts and tattoo images) that support the findings of this study are not publicly available due to their containing information that could compromise the privacy of research participants. De-identified data may be available from the corresponding author upon reasonable request and with appropriate ethical approval.

## Declarations

### Clinical trial registration

Clinical trial number: not applicable.
