# Supplementary material for: "This is the first time I felt they're mine": the significance of tattoos in coping and identity reconstruction following mastectomy
Source: Support Care Cancer. 2026 Aug 27;34(9):902. doi: 10.1007/s00520-026-11144-5 (PMC13521965; doi:10.1007/s00520-026-11144-5)
Supplement: Supplementary file 1 [file 520_2026_11144_MOESM1_ESM.pdf]

Note. The photographs included in this supplementary file were taken and submitted by the participants; none of the original images included identifiable facial features. The authors' only modification was cropping the photographs (e.g., to remove the neck) to reduce the surrounding background as much as possible while preserving the relevant visual content; no other digital editing or alteration was applied to the images.

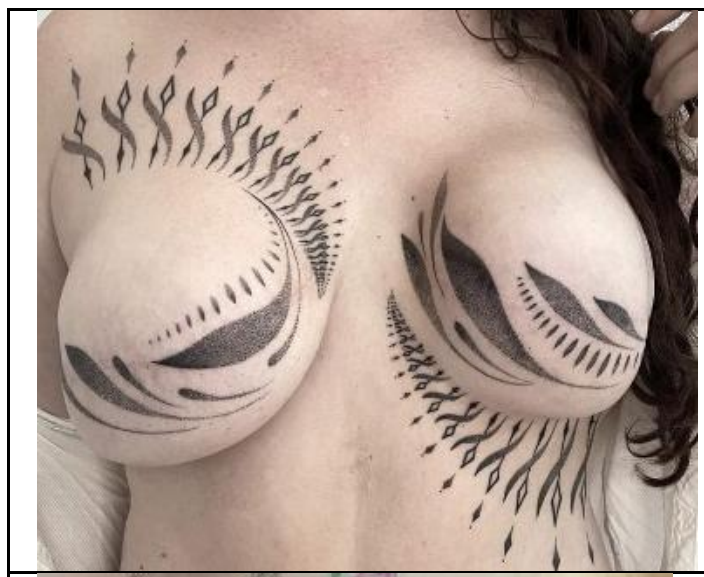	Shira<br>Geometric body flow, abstract
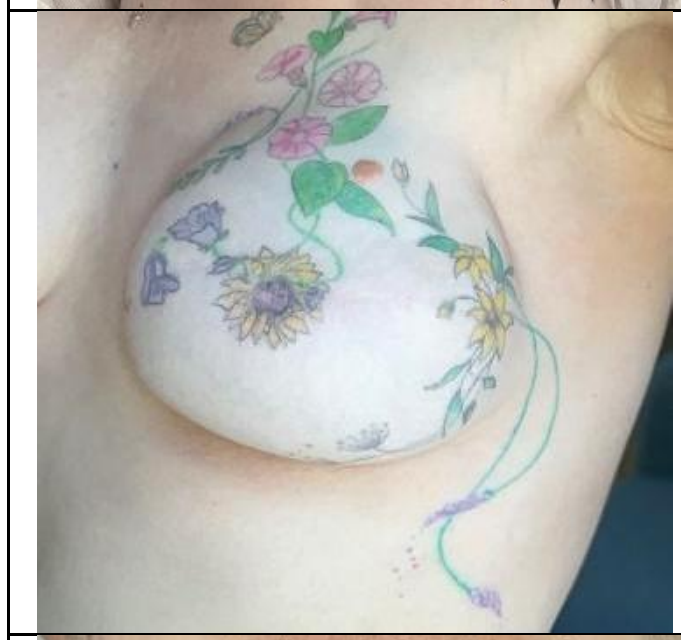	Rachel<br>Flowers,<br>sunflower<br>leaves, butterflies colorful
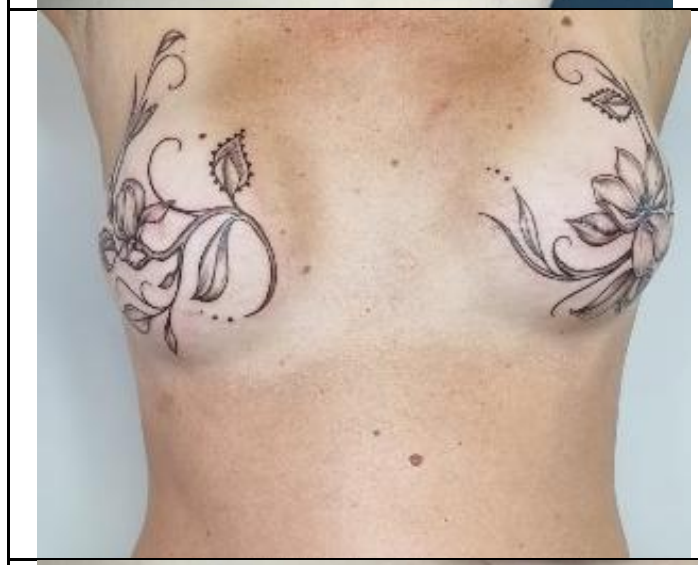	Eden<br>Flowers<br>leaves
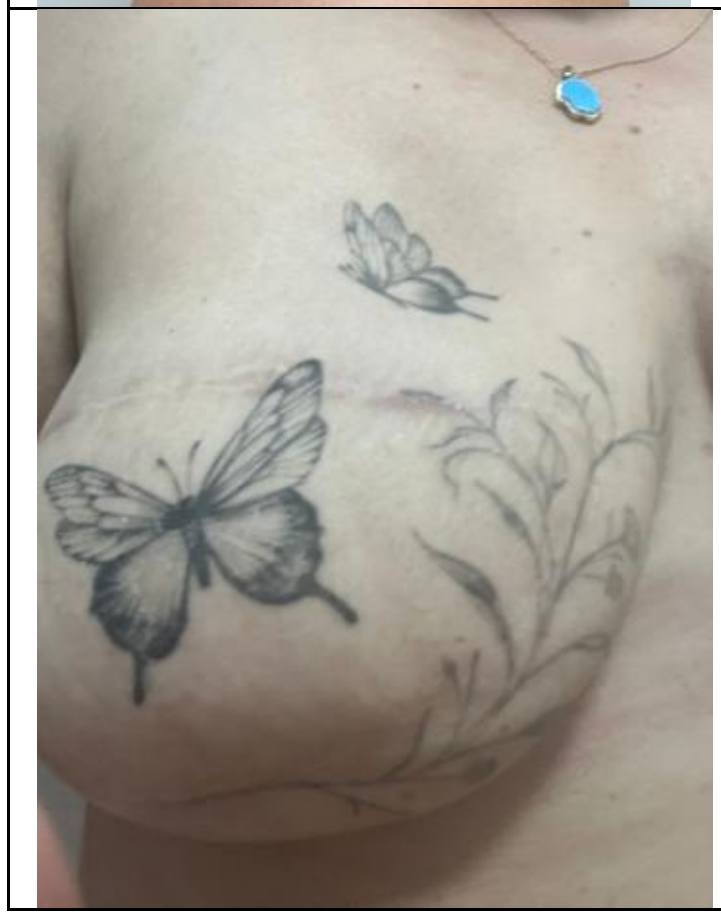	Neomi<br>Butterflies leaves

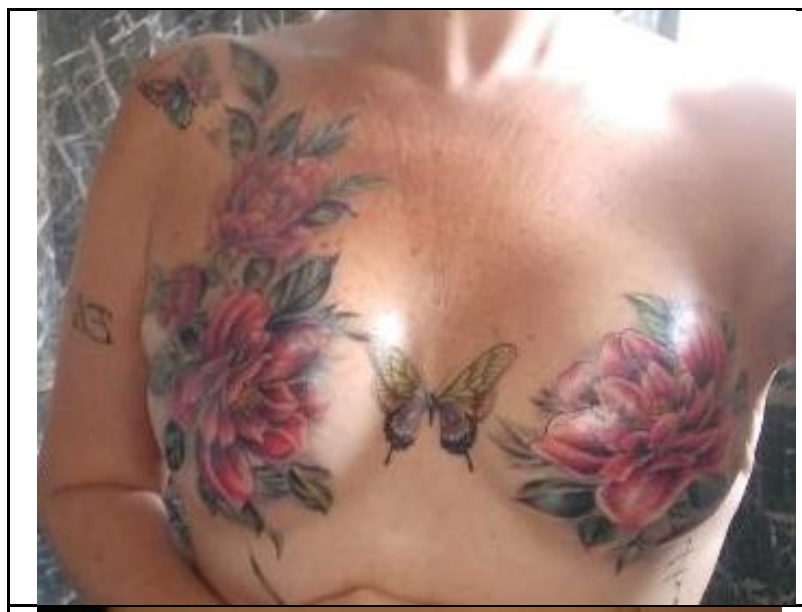	Miriam<br>Flowers, butterflies,<br>leaves colorful
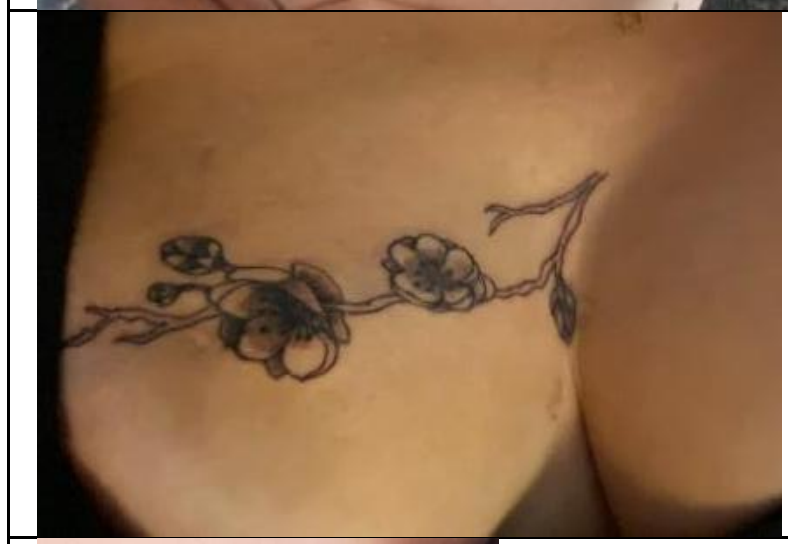	Dalia<br>Almond tree branch, flowers
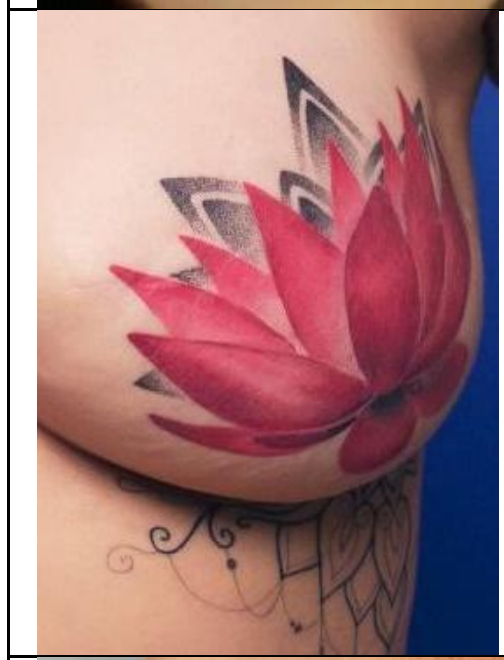	Ariel<br>Lotus flower<br>color
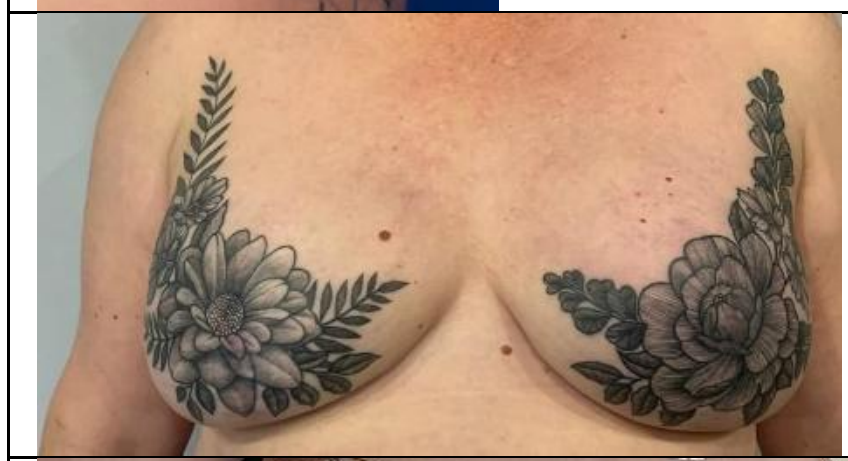	Abigail<br>Flowers, leaves
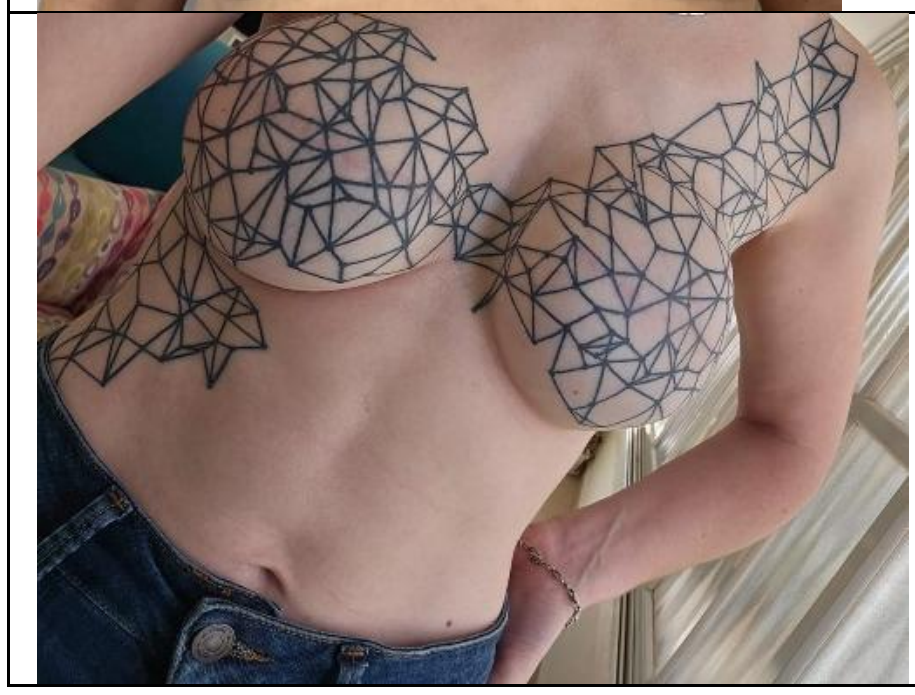	Michaela<br>Geometric figures, abstract

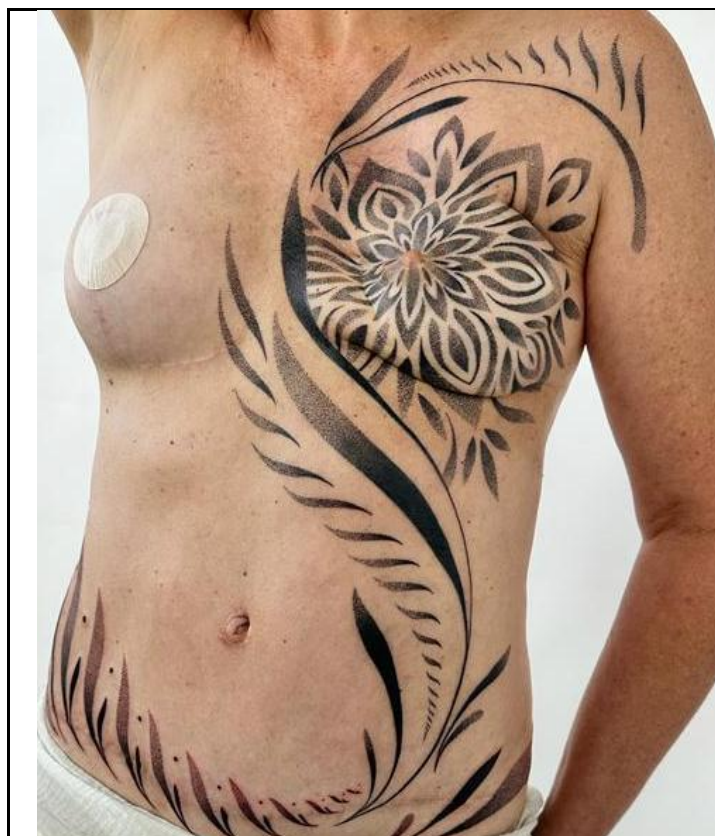	Maya<br>flower, abstract
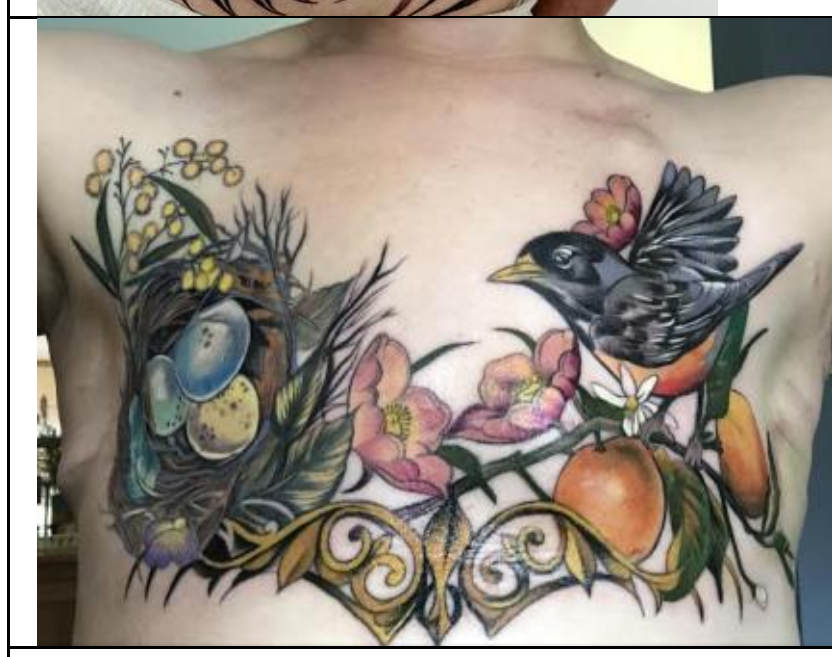	Jordan<br>Bird, fruit, flowers, nest, eggs<br>colorful
No available photo	Hannah
